# A Study on the Clustering Technology of Underwater Isomorphic Sensor Networks Based on Energy Balance

**DOI:** 10.3390/s140712523

**Published:** 2014-07-11

**Authors:** Fei Wang, Liming Wang, Yan Han, Bin Liu, Jian Wang, Xinyan Su

**Affiliations:** School of Information and Communication Engineering, North University of China, Taiyuan 030051, China; E-Mails: fayewang1987@foxmail.com (F.W.); hanyan@nuc.edu.cn (Y.H.); liubinnuc@126.com (B.L.); wangjian@nuc.edu.cn (J.W.); su_xy@nuc.edu.cn (X.S.)

**Keywords:** underwater sensors, UWHSN, UWISN, clustering, energy efficient, energy balance

## Abstract

Nowadays, there is a greater need for energy efficient and stable underwater sensor networks (UWSNs). Underwater sensors usually do not have enough power, so the goal of underwater sensor networks is to make the network have a long lifetime. An underwater heterogeneous sensor network (UWHSN) is one way to cluster the sensors, and the application of UWHSNs is simple and fast, but robots, lifetime and energy-partition are all drawbacks of UWHSNs. In this paper we propose the underwater isomorphic sensor network (UWISN) clustering technology. By analyzing the characteristics of UWISNs, we determine that an UWISN has strong expansibility, mobility, energy-efficiency and long lifetime. An UWISN adopts normal sensor nodes to be cluster heads, and these cluster heads communicate with each other. This paper seeks the optimal number of clusters and uses FCM to elect cluster heads and establish the network. In addition, an idea of real cluster heads and the method to elect them have been proposed. Finally, the simulation results show that the solution is effective and UWISNs can improve the energy consumption of an UWSN.

## 1. Introduction

In recent years, along with in-depth study of ocean development, underwater sensor networks have been established for military purposes, such as anti-submarine warfare, communications, positioning and guidance, in addition to increasing water resources position detection, underwater salvage, underwater environment warning [1]. An Underwater Sensor Network (UWSN) always uses the time schedule method, which has application in small scale UWSNs, but in massive UWSNs, the method above is inapplicable because of the high delay in acoustic links. Therefore, clustering methods have been introduced: the nodes near by the cluster head are divided into groups, and the others will join them.

Existing underwater sensor cluster networking technologies are mostly based on heterogeneous sensors, that is, the cluster heads are high energy nodes. The data of every cluster head has maximum load. An Underwater Heterogeneous Sensor Network (UWHSN) will effectively ease the energy depletion situation of the cluster heads and the network fast and easily [2,3]. However, there are also some inevitable drawbacks in such a method:

(1) Because the cluster heads are specified, when their energy has been exhausted, the robustness of an UWSN may be poor and network will be confronted with disruptions.
(2) Applications are limited, if there is full coverage of a huge area, there must be a huge number of sensors, including lots of high energy sensor nodes, and the cost will be increased.
(3) Sensor nodes will move with the ocean currents and wind, and their positions will change. Then, sensor nodes might be far away from their own cluster heads, and now the energy consumption of the network will increase. Therefore, the specified cluster heads will not absolutely be the best choice.

To figure out the UWHSN issues, this paper proposes an Underwater Isomorphic Sensor Network (UWISN) clustering technology. That is, the cluster heads are not specified, and in the initial stage every sensor node has the same energy and structure. According to algorithms, clustering technology of isomorphic sensors will periodically elect the best cluster heads on the basis of the current network environment, and then the rest of the nodes join in the clusters based on the principle of energy balance.

Advantages of the UWISN are:

(1) Without the permanent cluster heads, each node will have an opportunity to become a cluster head under the rule. When a cluster head dies, other nodes in the cluster will take over as the new cluster heads, and the sensor network lifetime is thus extended;
(2) Expansion ability will be enhanced, so when the nodes in the network are insufficient, a new node will enter the network to join the cluster immediately and also have permission to become a cluster head;
(3) Network mobility will be enhanced, whatever the effects of ocean currents or wind waves, so when sensors deviate from their original locations, they'll join the other clusters to work. If the cluster heads deviate, clusters will elect new cluster heads;
(4) The energy consumption of the network is distributed reasonably. One of the principles of cluster heads election is minimum energy consumption, therefore, the isomorphic sensor network lifetime cycle has been extended.

Compared with UWHSN, UWISN offers a more flexible network strategy and more reasonable networking rules.

## 2. Principle of Isomorphic Sensors Network Based on Energy Balance

Differing from UWHSNs using specified cluster heads, UWISNs just include normal sensor nodes distributed randomly in the monitoring region and one or more center nodes. In the UWISN, each node may be elected to be the cluster head; other nodes join the clusters according to the principle of energy-efficiency or to improve network quality [4].

This paper presents a study of isomorphic sensor networks based on energy balance. Depending on the determined optimal cluster size, optimal cluster heads with minimum energy consumption (referred to as optimal cluster heads) have been determined, and the remaining nodes join the clusters according to their distances to the cluster heads, and then become part of clusters as members. Because underwater sensor nodes are moving with the wind or wave movement, the locations of the determined optimal cluster heads are those of the optimal cluster heads only, finally, the real optimal cluster heads will be elected to construct clusters.

Above all, in order to improve networking flexibility and its achievement of energy balance, this paper proposes of clustering method for UWISNs:

(1) Depending on all randomly distributed sensor nodes which are isomorphic, N sensor nodes will be elected as cluster heads, whose residual energy is larger and whose distances from the central node are shorter, the others, as cluster members, communicate with the cluster heads in a single-hop method. N is the best number of clusters in this research;
(2) When N has been determined, the monitoring area will be covered by N clusters. The locations of theoretical cluster heads will be determined with algorithms, and then all the nodes will be assigned to the cluster heads. Clusters with theoretical optimal configuration will be constructed;
(3) After the 2nd step mentioned above, each of the sensor nodes distributes around a theoretical optimal cluster head location, after that, the real cluster heads should be elected. Underwater acoustic signals are attenuated with distance and frequency. Assuming the frequency is fixed, energy consumption only relates with distance. Therefore, nodes closer to the theoretical optimal cluster heads are going to be real optimal cluster heads more probably. Nodes elected as real cluster heads should satisfy following conditions: the shorter the distances *L_ic_* from the central nodes, the larger remaining energy of nodes *E_re_*/E_0_. The real cluster heads election formula is as follows:
  (1) $$U=w_{1}*L_{ic}+w_{2}*L_{\text{ice}}+(1-w_{1}-w_{2})(1-\frac{E_{re}}{E_{0}})$$
  When values of U are minimum, the corresponding nodes turn into cluster heads. In the above formula, *L_ice_* is an effect which means the values of distances from sensor nodes to the theoretical optimal cluster head, also, values of *L_ic_* are distances between nodes and theoretical optimal cluster head locations, and *w*_1_, *w*_2_ is weight values which are determined according to the network circumstances.
(4) After the real cluster heads have been elected, other nodes select one of them to construct clusters and join them. When a cluster head has been dead (the communication between cluster members and cluster head can't work), a new cluster head will be elected using the method above. When the amount of the living nodes isn't enough, new nodes should be added into the clusters. When it is necessary to enhance the coverage rate of a monitoring region, new nodes added immediately join the one of the fixed clusters according to their locations.

## 3. Study of Cluster Head Election Based on Energy Balance

The lifetime of underwater sensors is limited by their power. The power of the battery which nodes carry is limited, and when the power is exhausted and power endurance can't proceed, the validity of data transmission in sensor networks will be affected. Fundamental for cluster head election is that the average energy consumption of each cluster is minimum and the energy consumption of the whole network is minimum (also). This paper adopts a method to seek a minimum value of network energy consumption expectations, and then seek the cluster size. According to the known area of the motoring region, the number of cluster heads could be obtained in the background of minimum energy consumption.

Assuming that in cluster *x*, energy consumption of the member *i* to transport 1 bit of data to a cluster head with the method of single-hop communication, can be expressed as [5]:

(2) $$\left\{ \begin{array}{l} E_{xi}=E_{\text{elec}}+T\cdot C\cdot Hr_{xi}e^{\alpha (f)r_{xi}}\\ C=2\pi (0.67)10^{-9.5}\\ \alpha (f)=0.001\tilde{\alpha }(f)\ln10 \end{array}\right.$$

*r_xi_* is the distance between member *i* and cluster head *x*, *H* is the depth of *i* under the water, *T* is the signal propagation time, *α*(*f*) is the absorption coefficient of acoustic signal underwater.

After a cluster head has received the data of all nodes in the cluster, data fusion is going to proceed. The syncretic data will be transmitted to the central node by the cluster head. Energy consumption of cluster head ***x*** can be expressed as follows:

(3) $$E_{x}=nE_{\text{elec}}+n\cdot \eta (E_{\text{elec}}+T\cdot C\cdot Hde^{\alpha (f)d})$$

where *n* is the number of nodes in the cluster *x*. *η* is the amount of data which needs to be transmitted after data fusion, and its unit is bit. *d* is the distance between cluster head and central node. *E_elec_* is the energy consumption when the electronic equipment constructing sensor nodes transmits 1 bit of data.

After the energy consumption formula of cluster members and cluster heads has been obtained, the total energy consumption of the entire network can be expressed as follows:

(4) $$E_{\text{total}}=\sum_{x=1}^{N}(E_{x}+\sum_{i=1}^{n}E_{xi})$$

Assuming that the number of nodes contained by each of the clusters is the same, an expectation should be taken to *E_total_* which is in Formula (4), and then it can be expressed as follows:

(5) $${\bar{E}}_{\text{total}}=2NE_{\text{elec}}+N\cdot T\cdot C\cdot H\cdot E[re^{\alpha (f)r}]+N\cdot \eta (E_{\text{elec}}+T\cdot C\cdot H\cdot de^{\alpha (f)d})$$

It can be seen that the determinant of network total energy consumption is A = *re*^*α*(^*^f^*^)^*^r^* and B = *de*^*α*(^*^f^*^)^*^d^*. That is, the network total energy consumption is related to the node distribution. Assuming that each cluster has the same size, and then P represents the probability of nodes falling in the coverage of a cluster head, it can be expressed as:

(6) $$P(r)=\frac{\pi r^{2}}{\pi R_{c}^{2}}$$

*r* is the distance between nodes and the cluster head, *R_c_* is the radius of the cluster, which is a fixed value greater than the maximum value of *r*.

Assuming that monitoring region is a circular area whose radius is *R*, and probability means cluster heads falling in the area, it can be expressed as:

(7) $$P(r_{d})=\frac{\pi r_{d}^{2}}{\pi R^{2}}$$

From the above Formulas (6) and (7), the probability density function of *P*(*r*) and *P*(*r_d_*) can be sought, so the expectations of A and B can be expressed as:

(8) (8) $$E(A)=\int_{0}^{R_{c}}re^{\alpha (f)r}\frac{2r}{R_{c}^{2}}dr=\frac{2e^{\alpha (f)}R_{c}}{\alpha {\left(f\right)}^{3}}(\alpha {\left(f\right)}^{2}-\frac{2\alpha (f)}{R_{c}}+\frac{2}{R_{c}^{2}})-\frac{4}{\alpha {\left(f\right)}^{3}R_{c}^{2}}$$

(9) $$E(B)=\int_{0}^{R-R_{c}}r_{d}e^{\alpha (f)r_{d}}\frac{2r_{d}}{R_{c}^{2}}dr_{d}=\frac{2e^{\alpha (f)}(R-R_{c})}{\alpha {\left(f\right)}^{3}R^{2}}(\alpha {\left(f\right)}^{2}{\left(R-R_{c}\right)}^{2}-2\alpha (f))(R-R_{c})+2-\frac{4}{\alpha {\left(f\right)}^{3}R^{2}}$$

According to the Formulas (8) and (9), an expression of *Ē_total_* associated with cluster size *R_c_* can be obtained. Xiao seeks an extreme value of *Ē_total_* and when it is minimum, *R_c_* stands for the optimal number of clusters scale. The formula for seeking an extreme value is as follows:

(10) $$\begin{array}{l} \frac{\partial {\bar{E}}_{\text{total}}}{\partial R_{c}}=\frac{8+2e^{\alpha R_{c}(-4+4\alpha R_{c}-2\alpha^{2}R_{c}+\alpha^{3}R_{c}^{3})}}{\alpha^{3}Rc^{3}}+\eta \{\frac{1}{\alpha^{3}R^{2}}(e^{-\alpha R_{c}}(-4e^{\alpha R_{c}}+2e^{\alpha R}(\alpha^{2}R_{c}+\alpha (2-2\alpha R+\alpha R_{c}))))\}\\ -\frac{1}{\alpha^{2}R^{2}}(e^{-\alpha R_{c}}(-4e^{\alpha R_{c}}+2e^{\alpha R_{c}}(2+\alpha R(-2+\alpha R+\alpha Rc(2-2\alpha R+\alpha Rc))))) \end{array}$$

Keeping Formula (10) equal to 0 and adopting the Particle Swarm Optimization algorithm [6], *R_c_* could be sought under in different circumstances. The solutions of *R_c_* are shown in Table 1:

## 4. Formation of Clusters Based on Fuzzy C-Means Algorithm

When the optimal number of cluster heads has been determined, the set of all nodes *X{x_1_,x_2_,x_3_*……*x_N_}* will elect the optimal locations of cluster heads according to the value *c* which means the optimal number of cluster heads, and the nodes join the clusters by fuzzy membership. In the initial stage, the locations of cluster heads are in the center of clusters. According to the fuzzy c-means algorithm (FCM), we make the weight value of distances between cluster members and the cluster heads be minimum. It is shown as follows [7,8]:

(11) $$J_{m}(U,P)={\sum_{k=1}^{N}\sum_{i=1}^{c}\left(\mu_{ik}\right)}^{m}{\left(d_{ik}\right)}^{2}$$

(*d_ik_*)^2^ stands for the distances between cluster members and the cluster heads, and it is expressed as follows:

(12) $${\left(d_{ik}\right)}^{2}=\left\|x_{k}-p_{i}\right\|$$

Using the Lagrange multiplier method for solving, when *J_m_*(*U*, *P*) is obtained as the minimum value, *μ_ik_* and *p_i_* is expressed as follows:

(13) $$\mu_{ik}=\frac{1}{\sum_{j=1}^{c}{\left(\frac{d_{ik}}{d_{jk}}\right)}^{\frac{2}{m-1}}}$$

(14) $$p_{i}=\frac{\sum_{k=1}^{N}{\left(\mu_{ik}\right)}^{m}x_{k}}{\sum_{k=1}^{N}{\left(\mu_{ik}\right)}^{m}}$$

In the initial stage, *p_i_*^0^ is the location of cluster head. Set an iteration stopping threshold *δ*, by using an iteration method, and make ‖*P^t^*^+1^−*P*‖ ≤ *δ*. Finally, *p_i_* is the optimal location of cluster head [9,10]. Each of the nodes choose a cluster and join it according to *μ_ik_* which stands for fuzzy membership. Up to now, the clusters have been formed.

## 5. Simulation Results

This paper has used MATLAB to finish the numerical simulation. The simulation condition is R (radius of the monitoring region) = 500 m, N (the number of sensor nodes) =100 or 1000, and *η*= 0.5.

**Figure 1.**
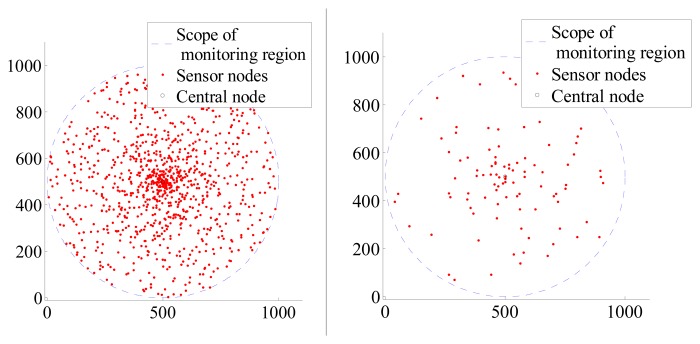
The left figure has 1000 sensor nodes. The right one has 100 sensor nodes.

When sensor nodes of different number distribute in the same monitoring region, It can be seen the distribution state in Figure 1.

**Figure 2.**
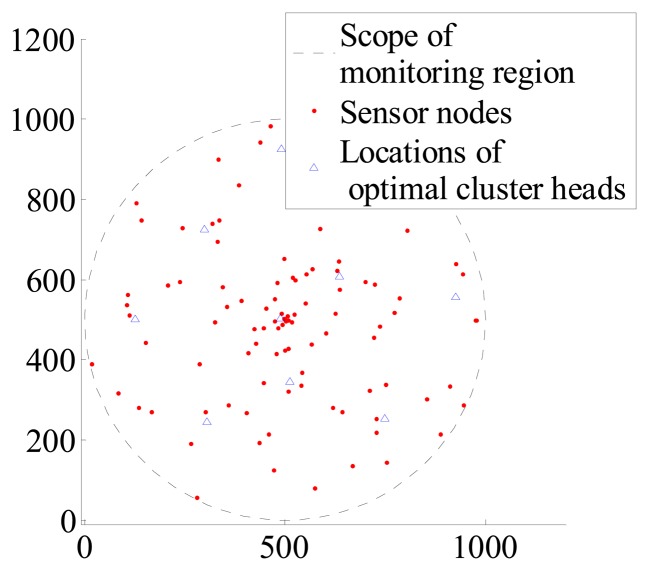
Distribution of optimal cluster heads when *c* = 9, N = 100.

**Figure 3.**
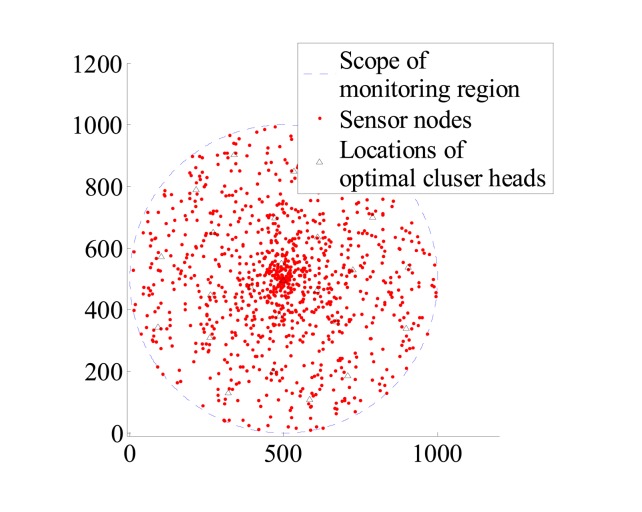
Distribution of optimal cluster heads when *c* = 25, N = 1000.

According to Section 3, *R_c_* has been sought. Therefore, the optimal number of cluster heads is $c=\frac{\pi R^{2}}{\pi Rc^{2}}$. Assuming that *c* = 9 and 25, the optimal locations of cluster heads have been sought in Section 4 and can be seen in Figures 2 and 3.

**Figure 4.**
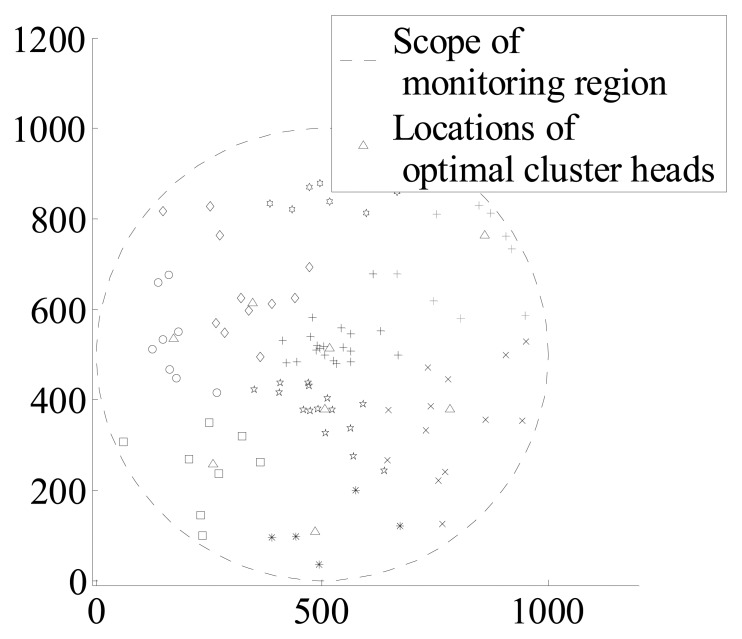
Distribution of clusters when *c* = 9, N = 100.

**Figure 5.**
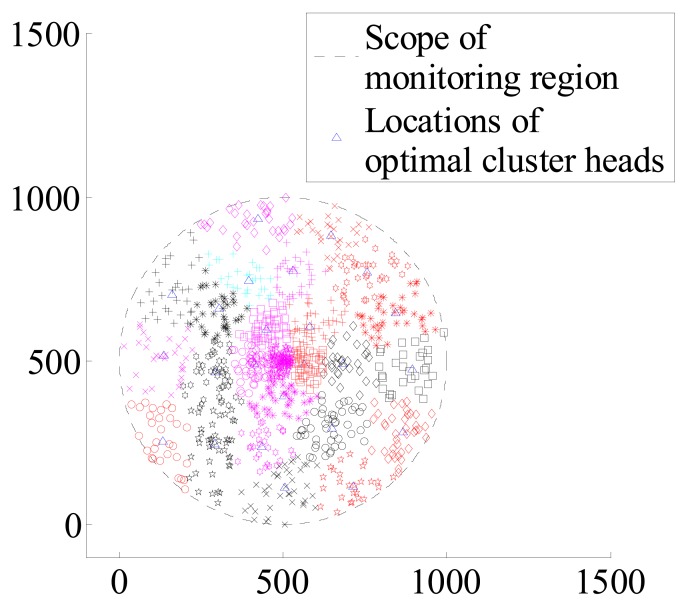
Distribution of clusters when *c* = 25, N = 1000.

According to the cluster membership *μ_ik_* of each sensor node, nodes belong to the cluster heads in which *μ_ik_* is maximum. Above all, clusters which have been formed can be shown in Figures 4 and 5.

Finally, all the clusters have been formed. Next the energy consumption of the entire network can be calculated. This paper just discusses the total consumption when N = 1000.

**Figure 6.**
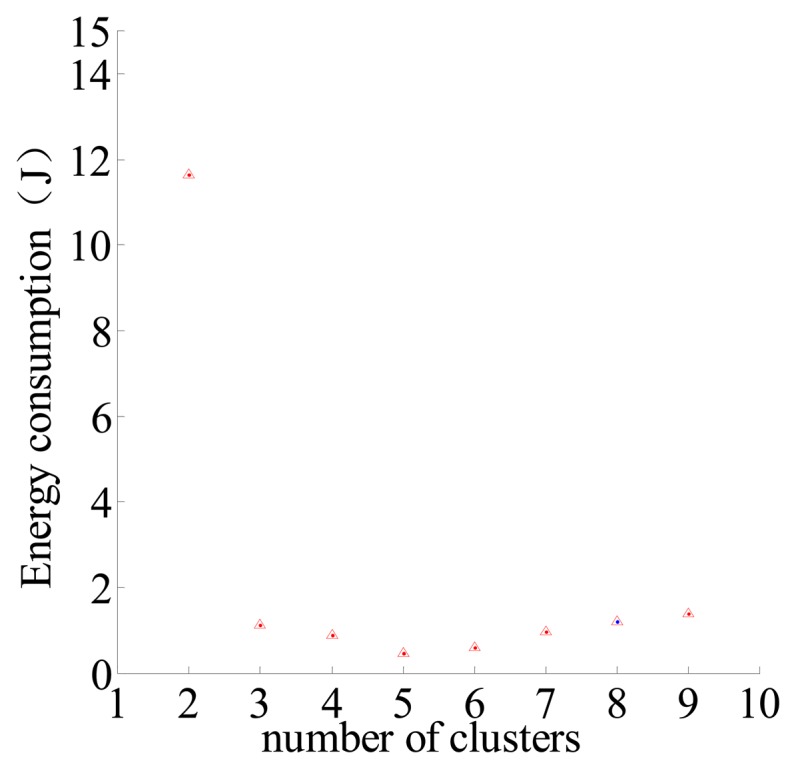
Different number of clusters-energy consumption relationship.

It can be seen in Figure 6, when *c* = 5, the energy consumption is minimum, and this result is almost the same as the result in Table 1 under the same conditions.

**Figure 7.**
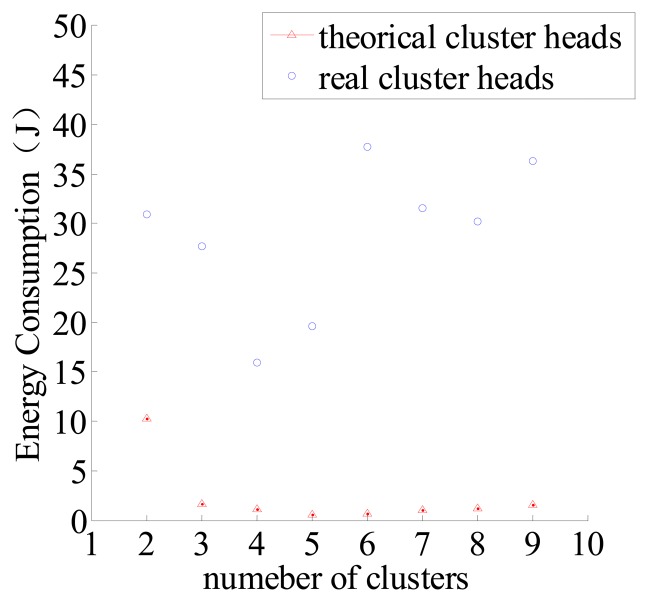
Different number of theoretical clusters and real clusters-energy consumption relationship.

Just as it has been discussed in Section 3, according to the locations of optimal cluster heads (theoretical cluster heads), it is necessary to elect the real cluster heads. Therefore, this paper has obtained the corresponding energy consumption of real cluster heads, and compared it with the theoretical cluster heads. This is shown in Figure 7.

It can be seen in Figure 7, when *c* = 4, the energy consumption using theoretical cluster heads is minimum, and when *c* = 5, the energy consumption using real cluster heads is minimum. That proves the result corresponding with real clusters is nearly the same as the theoretical clusters.

Energy consumption relates to the distribution of sensor nodes. The results in Figures 6 and 7 are the average results of 100 simulations, and the initial distribution of the nodes each time is not the same but it also conforms to a Poisson distribution.

## 6. Conclusions

In this study, we have explained UWISN and UWHSN and analyzed their characteristics. Then, we have researched the optimal cluster quantities according to the principle of UWISN based on energy balance. Adopting FCM, theoretical optimal cluster heads have been elected, and optimal clusters will have been formed. Because the probability of node distribution in the location of theoretical optimal cluster heads is quite small, we should elect real optimal cluster heads from the sensor nodes in the formed optimal clusters in fact. Finally, by the simulation, we have proved that the method to seek the quantities of optimal cluster heads and form optimal clusters is correct, and in addition, that the method to elect real optimal cluster heads is correct. Therefore, the UWISN based on energy balance is both energy efficient and feasible.

## Figures and Tables

**Table 1.** Solutions of *R_c_* in different circumstances.

*η*	*R***(m)**	*R_c_* **(m)**	**c (cluster number (int) *c* = π*R*^2^/π*R*_c_^2^)**
0.5	500	271	4
1.0	500	286	4
2.0	500	1082	1
0.5	1000	543	4
1.0	1000	521	4
2.0	1000	1072	1
0.5	2000	1170	4
1.0	2000	1062	4
2.0	2000	1097	4
